# Maternity continuum of care and its determinants among mothers who gave birth in Legambo district, South Wollo, northeast Ethiopia

**DOI:** 10.1002/hsr2.409

**Published:** 2021-11-02

**Authors:** Niguss Cherie, Mohammed Abdulkerim, Zinet Abegaz, Getaw Walle Baze

**Affiliations:** ^1^ Reproductive and Family Health Department School of Public Health, College of Medicine and Health Sciences, Wollo University Dessie Ethiopia; ^2^ Reproductive and Family health Legambo District Health Office South Wollo Ethiopia; ^3^ Epidemiology and Biostatics Department, School of Public Health College of Medicine and Health Sciences, Wollo University Dessie Ethiopia

**Keywords:** Ethiopia, Legamo district, maternity continuum of care

## Abstract

**Background:**

Maternity continuum of care is the continuity of maternity health care services that a woman uses for antenatal care, skill birth attendant, and postnatal care. Maternal and child mortality is still big challenge in Ethiopia. Little is known about continuum of maternity care in Ethiopia and where the study area in the district revealed that there is a big discrepancy in the completion of maternity care.

**Objective:**

Assessment of maternity continuum of care and associated factors among mothers who gave birth in Legambo district, South, Wollo, and northeast Ethiopia.

**Method:**

A community‐based cross‐sectional study design was conducted among 732 mothers from Feb‐Mar 2020. Multistage sampling was used and data were collected through face‐to‐face interviewer‐administered semi‐structured questionnaire. Completed data were entered using Epi‐Data version 3.1, cleaned, and analyzed using SPSS version 25 Statistical Software. Descriptive statistics using Frequency, proportion, summary measures were done. Binary logistic regressions were and model fitness was checked by Hosmer and Lemeshow test which was not significant. Multivariable logistic regression was conducted and *P* value less than .05 and adjusted odds ratio with 95% confidence interval was considered as statistically significant.

**Result:**

The prevalence of maternity continuum of care among mother was found 11.2% (95%, CI: 9.0‐13.8). Residence (AOR:1.837, CI:1.026‐3.288), planned pregnancy (AOR: 2.448, CI:1.361‐4.403), prepregnancy contraceptive utilization (AOR: 2.721, CI:1.469‐5.042), follow mass media (AOR: 2.33, CI:1.146‐4.736) and mother health care decision making autonomy (AOR: 3.712, CI:1.924‐7.161) were determinant factors to continuum of maternity care.

**Conclusion:**

The prevalence of maternity continuum of care in the district was low. Information education and counseling about continuum of care are still crucial. Awareness creation for both clients and care provider will improve the service. Efforts on improving and cultivating those significant factors should be done by stakeholders.

**Acronyms and Abbreviations**
ANC: antenatal care
AOR: adjusted odd ratio
BPCR: birth preparedness and complication readiness
CoC: continuum of care
COR: crud odd ratio
CSA: central statistics agency
EBR: Ethiopian Birr
FP: family planning
HEW: health extension worker
HIV: human immunodeficiency virus
MMR: maternal mortality ratio
MNCH: maternal neonatal and child health
PNC: postnatal care
SBA: skilled birth attendance
SPSS: statistical package for social sciences
WDA: women developmental army
WHO: World Health Organization

## 1 BACKGROUND

Maternity continuum of care is the continuity of maternity health care services that a woman uses the three recommended cares of antenatal care (ANC), skill birth attendant (SBA), and postnatal care (PNC).^1^ It is one of an important long‐term plan for reducing maternal and neonatal deaths and improving the health and well‐being of mothers and newborns.^1^, ^2^ The preventable causes of maternal and neonatal death can be reduced with provision of appropriate maternal health care services in a continuum manner.^3^, ^4^

Previous works focused on the continuum of care for child survival but continuum of care also much important for maternal health. A continuum of care links crucial interventions across the pregnancy, delivery, and postpartum stages.^4^, ^5^, ^6^ Adherence to continuum of care increase clients and health providers satisfaction as well help in increasing the efficiency of the service.^7^, ^8^ The advantages of continuum of care (CoC) are that each stage builds on the success of the previous stage.^9^, ^10^

Worldwide the overall figure regarding the magnitude of maternity continuum of care is not well known but some studies shows that it ranges from 60% in Cambodia to 27% in Pakistan.^11^, ^12^In Africa it ranges from 8% in Ghana to 31% in Nigeria.^13^, ^14^ Multi‐level study in 2019 showed that the magnitude of maternity continuum of care in Ethiopia is 9.1%.^15^

In 2017 Ethiopia has been considered as one of the 15 very high alert being fragile state high alert list countries^17^ with maternal mortality ratio (MMR) 412per 100 000 live births.^18^ According to Ethiopian demographic health survey (EDHS) women show some progress in maternal care utilization that at least four antenatal care from 32% to 43%, skilled delivery assistance from 28% to 50%, and postnatal care from 17% to 34% in EDHS 2016 and mini EDHS 2019 respectively.^18^, ^19^

Maternal and child mortality is still big challenge in Ethiopia. EDHS indicates progress in separate/fragmented service use of good ANC first visit but after that skilled delivery is not satisfactory and very low postnatal care service use. So, the aim of this study is continuum of care with our discontinuation of ANC, skill birth, and postnatal continuously and its determinates. Little is known about continuum of maternity care in Ethiopia and where the study area in the district revealed that there is big discrepancy on completion of maternity cares. Even though thus trends of reproductive health indicators from 2000 to 2019 showed that there is good progress of maternal health care services utilization, but the gap in the continuum of maternal health care services remains remarkably high.^20^

Therefore, this study aimed to assess the proportion of maternity continuum of care and its associated factors among mothers in Legambo district, South Wollo, Northeastern Ethiopia. The finding will help in identifying the prevalence of completion of maternity continuum of care and factors determining it and possible and fruitful intervention can be designed. The generated information will support to health programmers, policy makers in designing targeted health and related intervention programs; that lead to improve maternity care utilization.

## 2 METHOD AND MATERIALS

### 2.1 Study design, period, and area

Community‐based cross‐sectional study was conducted from February 25 to March 25, 2020. The study was carried out in Legambo District, South Wollo Zone, and Northeastern Ethiopia. Based on CSA in 2020 projection, the total population of the area had been projected to be 203 676 of which 50.38% were women. In the district, there are nine health centers one hospital and 11Private clinics. In the district, the annual estimated delivery of mothers was 6863 and half of it was be in 6 months according to Legambo district health office.^20^

### 2.2 Population and eligibility criteria

All women who gave birth and found in between first week to 6 months of postpartum period in Legambo district were source population. Those mothers who gave birth and found in between first week to 6 months of postpartum period in randomly selected kebeles were study population. Mothers who gave birth and found in between first week to 6 months after giving birth in the district before data collection were included and mothers who were not able to communicate due to known medical problems were excluded.

### 2.3 Sample size determination and procedure

Sample size had been estimated using single population proportion formula with the assumptions of 5% margin of error, 95% confidence interval, non‐response rate 10%, and 67.8% as a proportion maternity continuum of care completion in Debre Markos, Ethiopia.^23^ Sample size considering factors affecting maternity continuum of care were calculated using Epi Info version 7.3.2.1 using factors exposure to media, Place of residence and birth preparedness, and complication readiness.^15^, ^22^, ^23^ Sample size obtained from factor birth preparedness and complication readiness (n = 366) was the largest, then n = 366*2(design effect) n = 732 used as the final sample size. Multistage sampling method was conducted. First stage from 40 kebeles in the district 30% which were 12 kebeles had been selected by lottery method. Second stage from selected kebelles the individual mothers were selected using the list of mothers who delivered in each selected kebeles from the first week to 6 months of postpartum prior to data collection, then the sample size was distributed proportionally to each 12 kebeles. Study participants were selected randomly from the sampling frame from each selected kebeles.

### 2.4 Data collection tool and procedure

Data were collected through pre‐tested structured face‐to‐face interview. The structured questionnaires were prepared in a local language, Amharic, to make it simple and understandable. A total of five diploma midwives for data collection and one BSc public health officers as supervisors were involved. The data collector and the women developmental army were gone home to home. Mothers who were absent on the time of data collection were checked for the other day and mothers who were totally absent were replaced by the next neighbored mother.

### 2.5 Data quality control

The questionnaire was first prepared in English and then translated to Amharic and finally translated again to English by different individuals to check its translation accuracy and consistency. The trained data collectors were supervised during data collection and each questionnaire was checked for completeness on a daily basis. The questionnaire was pretested to check the response, languages clarity, and its appropriateness at Kelala district with (5%) of sample size.

### 2.6 Data management and analysis

Data were entered using Epi Data version 3.1 and exported to SPSS version 25.0 for analysis. Descriptive and analytical statics were done. Model fitting was checked by Hosmer and Lemshow test. Factors that show in bivariate analysis which had *P*‐value less than .25 were entered in to multivariable logistic regression models. The strength of statistical association had been measured by adjusted odds ratio, at 95% confidence intervals, and *P*‐value <.05 was considered as significant variable.

### 2.7 Operational definition

**Maternity continuum of care**: Women have four or more ANC visits by skilled provider plus Have had childbirth aided by skilled birth attendant (SBA) and Who attend postnatal care (PNC) three times at the health facility or by community health extension worker during their home visits within 24 hours,^26^, ^27^, ^28^ within 3 days,^27^, ^29^, ^30^, ^31^ and within a week.^27^

**Autonomy for health care decision‐making**: A woman said to have decision‐making power on seeking maternal health care service if she alone or with her husband decide on seeking service.^13^

**Women said to be exposed to media** if she Read a newspaper/listen to radio/watch television at least once a week are considered to be regularly exposed to that form of media.^18^

## 3 RESULT

### 3.1 Socio demographic characteristics

A total of 732 participants were enrolled in the study yields 100% response rate. The median age of the participants was 30 years it ranges between 18 and 44 years. from the study participants 464 (63.4%) were married and 547 (74.7%) were live in rural area (Table 1).

**TABLE 1:** Socio demographic characteristics of the mothers who gave birth in the last 6 months at Legambo district, South Wollo, northeast Ethiopia (n = 732)

Variables	Characteristics	Frequency	Present %
Age	Age below18‐19	55	7.5
	Age from 20‐34	424	57.9
	Age above 35	253	34.6
Marital status	Single	66	9
	Married	464	63.4
	Divorced	123	16.8
	Widowed	65	8.9
	Separated	14	1.9
Mothers education status	Cannot read and write	138	18.9
	Primary education (1–8)	429	58.7
	Secondary education (9–12)	137	18.6
	Tertiary (collage and above)	28	3.8
Mothers occupation	House wife	417	57
	Private employee	139	19
	Government employee	9	1.2
	Merchant	4	0.5
	Student	7	1
	Daily laborer	6	.8
	Farmer	150	20.5
Residence	Urban	185	25.3
	Rural	547	74.7
Husband education status	Cannot read and write	274	41.1
	Primary education (1–8)	181	27.2
	Secondary education (9–12)	124	18.6
	Tertiary (collage and above)	87	13.1
Husband occupation status	Private employee	72	9.8
	Government employee	86	12.9
	Merchant	212	31.8
	Student	12	1.8
	Daily labor	8	1.2
	Farmer	276	41.1
Head of household	Mother	163	22.3
	Husband	569	77.7

### 3.2 Autonomy and women's factor

Greater than half of the participants were used mass media and were not autonomies to make decision to get the health care as they need it. About 435 (59.4%) of the participants were not have information on the key pregnancy danger signs and 365 (49.9%) had not hear about the postnatal care service (Table 2).

**TABLE 2:** Women factors for mothers on maternity continuum of care in Legambo district, South Wollo, northeast Ethiopia (n = 732)

Variables	Characteristics	Frequency	Percent (%)
Follow mass media	Yes	395	54
	No	337	46
Type of media	Television	148	37.5
	Radio	210	53.2
	Social media	37	9.4
Far from district hospital	From 0.5 to 4 km	219	29.9
	From 5 to 9 km	164	22.4
	Above 9 km	349	47.7
Mother health care decision‐making autonomy	Yes	245	33.5
	No	487	66.5
Key pregnancy danger sign	Yes	297	40.6
	No	435	59.4
Thinking on childhood illness	All illness can be treated	146	19.9
	Some illness can be treated	300	41.0
	All illness cannot be treated	286	39.1
Heard about postnatal care	Yes	367	50.1
	No	365	49.9

### 3.3 Obstetric and maternity characteristics

The median ages of mothers at their first birth were 22 years and it ranges between 14 and 39 years. Greater than half of the participants did not plan for their last pregnancy. About three fourth of the participants did not us contraceptive before their last pregnancy and did not prepare for birth and for its complication if it happens (Table 3).

**TABLE 3:** Obstetric and maternity factors for mothers on maternity continuum of care in Legambo district, South Wollo, northeast Ethiopia (n = 732)

Variables	Characteristics	Frequency	Percent (%)
	Two	236	32.2
	Three	186	25.4
	Four and above	48	6.6
Planned pregnancy	Yes	310	42.3
	No	422	57.7
Age of mother at first birth	Age below 19	213	29.1
	Age from 20‐29	424	57.9
	Age above 30	95	13
Prepregnancy contraceptive use	Yes	202	27.6
	No	530	72.4
Birth preparedness and complication readiness	Yes	207	28.3
	No	525	71.7
Get counseling by health provider on family planning	Yes	277	37.8
	No	455	62.2

### 3.4 Proportion of maternity continuum of care

The prevalence of maternity continuum of care in the district was 11.2%; (95%, CI: 9.0‐13.8). According to this study skilled birth attendant was 33.9%, postnatal care service uptake was 13.2% and four and above ANC was 28.6%, Four ANC and skilled birth attendant together was 13.8%.

### 3.5 Determinant factors associated with maternity continuum of care

After excluding those variable with p value greater than 0.25 in bi variable analysis, mothers education, residence, planned pregnancy, prepregnancy, contraceptive utilization, birth preparedness and complication readiness, get counseling on family planning by health care provider, follow media, far from district hospital, mothers health care decision‐making autonomy, information on key pregnancy danger sign, thinking on childhood illness, heard about postnatal care, husbands education status, head of household, discuss on health issue with family member were entered to multivariable analyses. Multivariable analysis identified five independent factors affecting the maternity continuum of care. These were residence AOR:1.837 (CI:1.026‐3.288), planned pregnancy AOR;2.448 (CI:1.361‐4.403), prepregnancy contraceptive utilization AOR;2.721 (CI;1.469‐5.042), a follow mass media AOR;2.33 (CI;1.146‐4.736) and mother health care decision‐making autonomy AOR:3.712 (CI:1.924‐7.161) (Table 4).

**TABLE 4:** The bi variable and multivariable logistic regression result for factors associated for maternity continuum of care in Legambo district southeast Ethiopia (n = 732)

Variables	Category	Maternity continuum of care		COR(CI)	AOR(CI)
		Yes	No		
Residence	Yes	45	140	4.431 (2.76‐7.113)***	1.837 (1.026–3.288)*
	No	37	510	1	1
Planned pregnancy	Yes	57	253	3.578 (2.179‐5.874)***	2.448 (1.361–4.403)**
	No	25	397	1	1
Prepregnancy family planning use	Yes	59	143	9.095 (5.427‐15.24)***	2.721 (1.469–5.042)***
	No	23	507	1	1
Get counseling about family planning	Yes	48	229	2.595 (1.626‐4.144)***	1.724 (0.969‐3.067)
	No	34	421	1	1
Follow media	Yes	70	325	5.833 (3.103‐10.968)***	2.33 (1.146–4.736)*
	No	12	325	1	1
Mothers health care decision autonomy	Yes	61	184	7.357 (4.354‐12.429)***	3.712 (1.924–7.161)***
	No	21	466	1	1
Heard about PNC	Yes	64	303	4.072 (2.361‐7.023)***	1.811 (0.965‐3.398)
	No	18	347	1	1

Abbreviations: AOR, adjusted odd ratio; CI, confidence interval; COR, crude odd ratio.

* *P* < .05.

** *P* < .01.

*** *P* < .001.

## 4 DISCUSSION

This study assessed maternity continuum of care and associated factors among mothers who gave birth in Legambo District, South, Wollo Northeast Ethiopia. The overall prevalence of maternity continuum of care was 82 (11.2%) 95%, CI: 9.0‐13.8. It was consistence with the study done in Ghana (8%) and Tanzania (10%) from Africa.^13^, ^25^ and as well as it is consistence to the prevalence found in the study conducted in ArbaMinch (9.7%) and with the multilevel study conducted in Ethiopia which is (9.1%)^15^, ^22^ despite this the present study was smaller than the study done in Cambodia (60%) and Pakistan (27%).^11^, ^12^ This difference is because of that in sub‐Saharan Africa women and mothers live in poor accessibility to health institution and those health facilities are not well equipped that hinders mothers from using the services. This is also supported by study done on the determinates of women access to health care service in sub‐Saharan Africa.^26^

Besides, the prevalence in the present study was lower than study done in Debre Markos(67.8%)^23^ which might be due to, the proportion of mother with Tertiary(college and above) education was much higher in study done in Debre Markos (42.1%)^23^ than the present study (3.8%), for that matter the more educated the mother the more she can read and acquire knowledge and can balance the benefit of seeking health .this explanation also supported by Studies done in Pakistan in 2017, Lao PDR in rural Khammouane, in 2019, Southern Asia and Sub‐Saharan Africa in 2016, Nigeria in 2016, and Ghana in 2015, That Mothers who have higher level of education were 2.71 times more likely to get the maternity continuum of care than their counter parts respectively.^11^, ^13^, ^14^, ^21^, ^24^

And another explanation would be the difference in setting of study area in which study done in Debre Markos was in urban town which is the zonal capital of East Goijam Zone, whereas the present study was from rural kebeles dominated wereda, in which greater than 74% live in rural kebele that the access of health facility was compromised to access in rural weredas and this explanation also supported by the current study, study in Pakistan in 2017 and Southern Asia and sub‐ Sahara Africa in 2016 that mothers in urban area where1.8,1.2 and 2.3 times more likely to complete the maternity continuum of care than their counter parts respectively.^11^, ^21^

In the present study mothers who planned their pregnancy were 2.4 times more likely have completion of maternity care compared to non‐planed. Which is in line with study conducted in Ghana as well as studies conducted in Ethiopia in Arbaminch zuria wereda 2019 and Debremarkos town 2019 in which mothers who plan their pregnancy were 1.75, 3.4, 3.4times more likely had completion of maternity continuum of care than their counter parts respectively.^18^, ^19^ This can be explained by as, in this study, more than 50% of unmarried (not in union) women's pregnancies were unplanned, so that they might not accept their pregnancies to themselves and hide it to others because mostly pregnancies without marriage were highly stigmatized.^27^and which is supported by study conducted in Ghana in 2015 that those married were 69%more likely to attend the maternity continuum of care than their counter parts.^13^

In the present study mothers who follow media were 2.3 times more likely to get the maternity continuum of care than their counterparts. And this is in line with the study done in Pakistan 2017 and Ethiopia in Debre markos town 2019 in which mothers who follow media were1.45 and 2.62 times more likely to complete the maternity continuum of care respectively.^11^, ^23^ This can be explained as mothers who have access to media can get more information and promotion on maternal health care service that also brief the services that can be accessed to the nearby health institutions which encourage the mother use and be familiar to the maternal health services. This explanation is supported by the study done in South Asia 2019 on mass media exposure and maternal healthcare utilization in which Women exposed to mass media were 39% to 113% more likely to receive antenatal care, 17% to 99% more likely to deliver their babies by skilled birth attendants, and 24% to 95% more likely to receive postpartum check‐ups after their delivery across countries.^28^

In this study Mothers who used prepregnancy contraceptives were 2.7 times more likely to complete maternity care compared to their counter parts. it is consistence with the study done in Arbaminch zurai wereda that mothers who us prepregnancy contraceptive were3.9 times more likely to get the maternity continuum of care than their counter parts.^15^, ^22^, ^23^ The reason behind might be women's who utilized prepregnancy contraception were well informed about the next maternal and newborn services and set plans with health professional for next services. This explanation also supported by findings from a systematic review and meta‐analysis on preconception care where the odds of attending antenatal care for women who were counseled about subsequent services during preconception period were 39% higher when compared to their counter parts.^29^

In the current study mothers who were autonomies in health care decision‐making were 3.7 times more likely to complete the maternity continuum of care compared to their counter parts. Which is in line with study done in Pakistan in which mothers who were autonomies in health care decision were 1.26 times more likely to complete the continuum of care compared to their counter parts.^11^ This can be explained as that mothers who are autonomies on health decision are free to seek care for their health, which they do not need to wait someone else to decide on her health issue. and this explanation also supported by study done on women's autonomy in health care decision‐making in developing countries.^30^

## 5 LIMITATION OF THE STUDY

Social desirability and recall bias, maybe introduced in the time of data collection and temporal relation between dependent and independent variable cannot be established since it is cross sectional study.

## 6 CONCLUSION AND RECOMMENDATION

The prevalence of maternity continuum of care in the district was relatively low. Residence, planned pregnancy, prepregnancy contraceptive utilization, mass media follow, and autonomy of the mother on health care decision‐making affect the maternity continuum of care. Awareness creation for both clients and care provider will improve the service. Ministry of health and concerned stakeholders should work on the accessibility of health service in rural areas and decision‐making autonomy of the mothers to be uplifted through educating and empowering women, District health office should disseminate the information and counseling aiming the accessibility, availability, benefit, and the services offered on the health facilities on maternal health. Further studies with qualitative methods of cultural factors, barriers, and facilitators of continuum of care investigation are recommended to up lift the status of maternity continuum of care.

## FUNDING

This research project was funded by Wollo University. The funder has no role in publication process.

## CONFLICT OF INTEREST

The authors declare that there are no conflicts of interest regarding the publication of this paper.

## TRANSPARENCY STATEMENT

The lead author (Niguss Cherie) affirms that this manuscript is an honest, accurate, and transparent account of the study being reported; that no important aspects of the study have been omitted; and that any discrepancies from the study as planned have been explained.

## AUTHOR CONTRIBUTIONS

Conceptualization: Niguss Cherie, Mohammed Abdulkerim, Zinet Abegaz, Getaw Wale

Data Curation: Mohammed Abdulkerim

Data integrity: Mohammed Abdulkerim.

Formal Analysis: Niguss Cherie, Mohammed Abdulkerim

Methodology: Niguss Cherie, Zinet Abegaz, Getaw Wale

Software: Mohammed Abdulkerim

Supervision: Niguss Cherie, Getaw Wale

Writing—Original Draft Preparation: Niguss Cherie, Zinet Abegaz, Getaw Wale

Writing—Review and Editing: Niguss Cherie, Mohammed Abdulkerim, Getaw Wale

All authors have read and approved the final version of the manuscript.

## ETHICS STATEMENT

Ethics approval and informed consent: Ethical and aware verbal consent was approved ethical review board of Wollo University, school of medication and Health Sciences. The study complies with the Declaration of Helsinki and fulfills moral assumptions. Every study participant was communicated clearly concerning the target of the study to get their consent, their full right to withdraw or refuse to participate. Privacy was kept during data collection.

## Data Availability

The data supporting the findings of this study are available from the corresponding author upon request. The corresponding author had full access to all of the data in this study and takes complete responsibility for the integrity of the data and the accuracy of the data analysis.
